# Supplementary material for: Prediction of enzymatic pathways by integrative pathway mapping
Source: eLife. 2018 Jan 29;7:e31097. doi: 10.7554/eLife.31097 (PMC5788505; doi:10.7554/eLife.31097)
Supplement: Supplementary file 1. — Three well characterized pathways were used for benchmarking of the integrative pathway mapping: glycolysis (10 enzymes) (Kalyanaraman and Jacobson, 2010), cytidine monophosphate 3-deoxy-D-manno-octulosonate 8-phosphate (CMP KDO-8P) biosynthesis (four enzymes), and serine biosynthesis (five enzymes). [file elife-31097-supp1.docx]

|  | **Glycolysis pathway,**  ***E. coli*** | **CMP KDO-8P pathway** | **Serine biosynthesis pathway** |
| --- | --- | --- | --- |
| **# of enzymes** | 10 | 4 | 5 |
| **# of metabolites** | 2,965 | 3,336 | 3,494 |
| **Docking program** | Glide and MM-GBSA scoring  (Kalyanaraman and Jacobson, 2010) | DOCK | DOCK |
| **Monte Carlo sampling runs** | 1,000 | 2,000 | 2,000 |
| **Number of steps per run** | 5,000,000 | 500,000 | 500,000 |
| **Number of unique pathways sampled** | 140,558,544 | 12,258,665 | 58,804,323 |
| **Rank of correct pathway** | 1 | 18 | 1 |
| **Number of top-scoring pathways** | 1,100 | 29 | 91 |
| **Number of Clusters** | 3 | 5 | 5 |
