# Supplementary material for: Prediction of enzymatic pathways by integrative pathway mapping
Source: eLife. 2018 Jan 29;7:e31097. doi: 10.7554/eLife.31097 (PMC5788505; doi:10.7554/eLife.31097)
Supplement: Supplementary file 2. — The number of candidate ligands as input is 3650 for the L-gulonate catabolic pathway. The ranks from individual docking runs are determined by docking score for an enzyme. The ranks by the integrative approach are determined from the relative frequency of ligand-enzyme pairs in the ensemble of high-scoring pathway models. This frequency is then used to order ligands for a given enzyme. [file elife-31097-supp2.docx]

| **Enzyme** | **Docking rank from individual run** | | | **Integrative approach rank** | |
| --- | --- | --- | --- | --- | --- |
|  | **Substrate** | | **Product** | **Substrate** | **Product** |
| ***Glycolysis benchmark pathway*** | | | | | |
| 1) Glucokinase | 37 | | 1354 | 3 | 1 |
| 2) Phosphoglucose isomerase | 108 | | 77 | 1 | 1 |
| 3) Phosphofructokinase | 18 | | 30 | 1 | 2 |
| 4) Fructose bisphosphate aldolase | 183 | | 158 | 2 | 1 |
| 5) Triosephosphate isomerase | 12 | | 130 | 1 | 1 |
| 6) Glyceraldehyde 3-phosphate dehydrogenase | 865 | | 2918 | 1 | 1 |
| 7) Phosphoglycerate kinase | 71 | | 74 | 1 | 1 |
| 8) Phosphoglycerate mutase | 5 | | 2 | 1 | 1 |
| 9) Enolase | 27 | | 76 | 1 | 1 |
| 10) Pyruvate kinase | 129 | | 898 | 1 | 1 |
| ***CMP-KDO-8P synthesis benchmark pathway*** | | | | | |
| 1) D-arabinose 5-phosphate isomerase | 3228 | 3238 | | 1 | 1 |
| 2) KDO-8P synthase | 543 | 36 | | 2 | 3 |
| 3) KDO-8P Phosphatase | 3204 | 2804 | | 4 | 5 |
| 4) CMP-KDO synthase | 1607 | 3001 | | 1 | 1 |
| ***Serine/cysteine biosynthesis benchmark pathway*** | | | | | |
| 1) 2-hydroxyglutaric acid dehydrogenase | 3351 | 3276 | | 1 | 1 |
| 2) 3-phosphoserine aminotransferase | 9 | 6 | | 1 | 1 |
| 3) Phosphoserine phosphatase | 4 | 250 | | 1 | 1 |
| 4) Serine acetyltransferase | 913 | 806 | | 1 | 1 |
| 5) O-acetylserine lyase | 2003 | 1472 | | 1 | 1 |
| ***Prospective case: L-gulonate catabolic pathway*** | | | | | |
| 1) GulD | 1752 | 1114 | | 1 | 1 |
| 2) UxuB | 2976 | 3378 | | 1 | 1 |
| 3) UxuA | 387 | 55 | | 1 | 1 |
| 4) KdgK | 1187 | 3540 | | 1 | 1 |
| 5) KdgA | 33 | 7 | | 1 | 1 |
