# Supplementary material for: Prediction of enzymatic pathways by integrative pathway mapping
Source: eLife. 2018 Jan 29;7:e31097. doi: 10.7554/eLife.31097 (PMC5788505; doi:10.7554/eLife.31097)
Supplement: Supplementary file 3. — The ΔTm for each ligand was calculated as the difference of the Tm values measured with a ligand and without ligand for SBS from Haemophilus influenzae RdAW and Mannheimia haemolytica PHL213 (GulP: 82% sequence identity). [file elife-31097-supp3.docx]

|  | **Δ*T*_m_  of TRAP solute binding protein (Temperature in °C)** | |
| --- | --- | --- |
| **Ligand Name** | ***Hi*GulP**  **(Uniprot ID P71336; PDB ID 4PBQ)** | ***MHH*GulP**  **(Uniprot ID A7JQX0)** |
| **L-gulonate** | 10.9 | 12.1 |
| **D-mannonate** | 7.3 | 8.2 |
| **L-idonate** | 2.1 | - |
| **L-galactonate-6P** | 2.95 | 4.15 |
