# Supplementary material for: Prediction of enzymatic pathways by integrative pathway mapping
Source: eLife. 2018 Jan 29;7:e31097. doi: 10.7554/eLife.31097 (PMC5788505; doi:10.7554/eLife.31097)
Supplement: Supplementary file 4. [file elife-31097-supp4.docx]

| **KEGG ID** | **Name** |
| --- | --- |
| C00022 | Pyruvate |
| C00024 | Acetyl-CoA |
| C00026 | 2-Oxoglucarate |
| C00036 | Oxaloacetate |
| C00042 | Succinate |
| C00074 | Phosphoenolpyruvate |
| C00091 | Succinyl-CoA |
| C00111 | Glycerone phosphate |
| C00117 | D-Ribose 5-phosphate |
| C00118 | D-Glyceraldehyde 3-phosphate |
| C00122 | Fumarate |
| C00149 | (S)-Malate |
| C00158 | Citrate |
| C00197 | 3-Phospho-D-glycerate |
| C00199 | D-Ribulose 5-phosphate |
| C00231 | D-Xylulose 5-phosphate |
| C00236 | 3-Phospho-D-glyceroyl phosphate |
| C00267 | alpha-D-Glucose |
| C00279 | D-Erythrose 4-phosphate |
| C00311 | Isocitrate |
| C00345 | 6-phospho-D-gluconate |
| C00631 | 2-Phosphoenolpyruvate |
| C00668 | alpha-D-Glucose 6-phosphate |
| C01172 | beta-D-Glucose 6-phosphate |
| C01236 | D-Glucono-1,5-lactone 6-phosphate |
| C05345 | beta-D-Fructose 6-phosphate |
| C05378 | beta-D-Fructose 1,6-bisphosphate |
| C05382 | Sedoheptulose 7-phosphate |

# 
