# Supplementary material for: Prediction of enzymatic pathways by integrative pathway mapping
Source: eLife. 2018 Jan 29;7:e31097. doi: 10.7554/eLife.31097 (PMC5788505; doi:10.7554/eLife.31097)
Supplement: Supplementary file 5. [file elife-31097-supp5.docx]

|  | ***Glycolysis benchmark pathway*** | | | |
| --- | --- | --- | --- | --- |
| **Steps in pathway** | **Enzyme** | **EC Number** | **Structure/**  **template**  **(GI number)** | **SMIRKS Reactions** |
| **1** | Glucokinase | 2.7.1.2 | 1SZ2 (16130320) | [C:1][O:2][H]>>[C:1][O:2][P]([O])([O])=O |
| **2** | Phosphoglucose isomerase | 5.3.1.9 | 2CXR (16131851) | [O:3][C:2][C:1][H]>>[O:3][C:1][C:2][H] |
| **3** | Phosphofructokinase | 2.7.1.11 | 1PFK (16131754) | [C:1][O:2][H]>>[C:1][O:2][P]([O])([O])=O |
| **4** | Fructose bisphosphate aldolase | 4.1.2.13 | 3ELF (16130826) | [H][O:1][C:2](A)[C:3][O:4]A>>[O:1]=[C:2][C:3][O:4][H]  A[C:1]([O:2][H])[C:3]([O:4][H])A>>[O:2]=[C:1][C:3]([O:4][H])[H] |
| **5** | Triosephosphate isomerase | 5.3.1.1 | 7TIM (16131757) | [H][O:1][C:2]([H])[C:3](=[O:5])[C:4]>>[O:1]=[C:2][C@:3]([H])([O:5][H])[C:4]  [H][O:1][C:2]([H])[C:3](=[O:5])[C:4]>>[O:1]=[C:2][C@@:3]([H])([O:5][H])[C:4] |
| **6** | Glyceraldehyde 3-phosphate dehydrogenase | 1.2.1.12 | 1NQA (16129733) | [O:2]=[C:1][H]>>[O:2]=[C:1][O][P](O)(O)=O |
| **7** | Phosphoglycerate kinase | 2.7.2.3 | 1VPE (16130827) | [C:1][O:2][P]([O])([O])=O>>[C:1][O:2] |
| **8** | Phosphoglycerate mutase | 5.4.2.11 | 1EJJ (16131483) | [O:5][P:6]([O:7])(=[O:8])[O:4][C:1][C:2][O:3][H]>>[H][O:4][C:1][C:2][O:3][P:6]([O:5])([O:7])(=[O:8]) |
| **9** | Enolase | 4.2.1.11 | 1EBG (16130686) | [H][C:1][C:2][O][H]>>[C:1]=[C:2] |
| **10** | Pyruvate kinase | 2.7.1.40 | 1AQF (16139632) | [C:1]=[C:2][O:3][P]([O])([O])=O>>[H][C:1][C:2]=[O:3] |
|  | ***CMP-KDO-8P synthesis benchmark pathway*** | | | |
| **Steps in pathway** | **Enzyme** | **EC Number** | **Structure (Sequence homologues used for docking)** | **SMIRKS Reactions** |
| **1** | D-arabinose 5-phosphate isomerase | 5.3.1.13 | 2XHZ/3ETN | [H][O:1][C:2]([H])[C:3]=[O:4]>>[O:1]=[C:2][C:3]([H])[O:4][H] |
| **2** | KDO-8P synthase | 2.5.1.55 | 4LU0 | [C:1]=[O:2]>>[C:1]([O:2][H])CC(=O)C(=O)O |
| **3** | KDO-8P Phosphatase | 3.1.3.45 | 3E81/4HGP | [C:1][O:2][P]([OH])([OH])=O>>[C:1][O:2]  [C:1][O:2][P]([O;X1])([O;X1])=O>>[C:1][O:2] |
| **4** | CMP-KDO synthase | 2.7.7.38 | 1H7T | [H][O:1][C:2][A:5][A:6][A:7][C:3]=[O:4]>>[O:1]1[C:2][A:5][A:6][A:7][C:3]1[O:4]P(=O)(OC[C@H]1O[C@H]([C@@H]([C@@H]1O)O)n1c(=O)nc(cc1)N)O |
|  | ***Serine biosynthesis benchmark pathway*** | | | |
| **Steps in pathway** | **Enzyme** | **EC Number** | **Structure (Sequence homologues used for docking)** | **SMIRKS Reactions** |
| **1** | 2-hydroxyglutaric acid dehydrogenase | 1.1.1.95 | 1PSD/3DDN | [H][C:1][O:2][H]>>[C:1]=[O:2] |
| **2** | 3-phosphoserine aminotransferase | 2.6.1.52 | 1W23 | [C:1]=[O]>>[H][C:1][NH2] |
| **3** | Phosphoserine phosphatase | 3.1.3.3 | 1F5S | [C:1][O:2][P]([OH])([OH])=O>>[C:1][O:2][H] |
| **4** | Serine acetyltransferase | 2.3.1.30 | 3GVD/1SST | [C:1][O:2][H]>>[C:1][O:2][C]([CH3])=O |
| **5** | O-acetylserine lyase | 2.5.1.47 | 3VC3/3VSD | [C:1][O][C]([CH3])=O>>[C:1][S][H] |
