# Supplementary material for: Prediction of enzymatic pathways by integrative pathway mapping
Source: eLife. 2018 Jan 29;7:e31097. doi: 10.7554/eLife.31097 (PMC5788505; doi:10.7554/eLife.31097)
Supplement: Supplementary file 6. [file elife-31097-supp6.docx]

| **Enzyme (Uniprot ID)** | **Protein name** | **Locus ID** | **Structure (Template PDB ID)** | **SMIRKS** |
| --- | --- | --- | --- | --- |
| **Q57517** | *Hi*GulD | HI0053 | 4ILK | [H][O:1][C:2]([H])([A:3])[A:4]>>[O:1]=[C:2]([A:3])([A:4])  [O:1]=[C:2]([A:3])([A:4])>>[H][O:1][C:2]([H])([A:3])[A:4] |
| **P44481** | *Hi*UxuB | HI0048 | 3GAF | [O:1]=[C:2]([A:3])([A:4])>>[H][O:1][C@:2]([H])([A:3])[A:4]  [H][O:1][C@:2]([H])([A:3])[A:4]>>[O:1]=[C:2]([A:3])([A:4]) |
| **P44488** | *Hi*UxuA | HI0055 | 4EAY | [H][O:1][C:2]([H])[C:3]([H])[O][H]>>[O:1]=[C:2]([H])[C:3]([H])[H]  [O:1]=[C:2]([H])[C:3]([H])[H]>>[H][O:1][C:2]([H])[C:3]([H])[O][H] |
| **P44482** | *Hi*KdgK | HI0049 | 3LHX | [C:1][O:2][H]>>[C:1][O:2][P](O)(O)=O  [C:1][O:2][P](O)(O)=O>>[C:1][O:2][H] |
| **P44480** | *Hi*KdgA | HI0047 | 1VHC | [A:1][C:2][C:3]([O:4][H])[A:5]>>[A:1][C:2][H].[O:4]=[C:3][A:5] |
| **P71336** | *Hi*GulP | HI0052 | 2XWK |  |
