# Supplementary material for: Prediction of enzymatic pathways by integrative pathway mapping
Source: eLife. 2018 Jan 29;7:e31097. doi: 10.7554/eLife.31097 (PMC5788505; doi:10.7554/eLife.31097)
Supplement: Supplementary file 7. [file elife-31097-supp7.docx]

| **Gene name (Uniprot #)** | **Primer name** | **Sequence** |
| --- | --- | --- |
| ***Hi*UxuB**  **(Uniprot ID P44481)** | HiFR_FOR | 5’- TACTTCCAATCCATGGAGTTTACTATGAATATTGCAGCAAACC -3’ |
|  | HiFR_REV | 5’- TATCCACCTTTACTGTTAGACTCCACTATACGCAGAAAAACC -3’ |
| ***Hi*KdgK**  **(Uniprot ID P44482)** | HiKdgK_FOR | 5’- TACTTCCAATCCATGAAAAAAATAGCATTTATCGGCGAGTGTATG -3’ |
|  | HiKdgK_REV | 5’- TATCCACCTTTACTGTTAATTAAATTCAGATTGAAGATGAGAAATTGCG -3’ |
| ***Hi*KdgA**  **(Uniprot ID P44480)** | HiKdgpA_FOR | 5’- TACTTCCAATCCATGTCATACACAACTCAACAAATTATTGAAAAACTTC -3’ |
|  | HiKdgpA_REV | 5’- TATCCACCTTTACTGTTATTTAATAATATCAATCACTTCTCTGACTAATCG -3’ |
| ***Hi*GulD**  **(Uniprot ID Q57517)** | HiGulDH_FOR | 5’- TACTTCCAATCCATGAAGGAGCGAATCAAAGCAATATGTTTAG -3’ |
|  | HiGulDH_REV | 5’- TATCCACCTTTACTGTTAGAAAACTGCATTAATTTTTAAATAATTTTCTGGATG -3’ |
| ***Hi*UxuA**  **(Uniprot ID P44488)** | HiUxuA_FOR | 5’- TACTTCCAATCCATGGAACAAGCATGGCGTTGGTATGG -3’ |
|  | HiUxuA_REV | 5’- TATCCACCTTTACTGTTACTTATTGAAATACACTTTTTTTAATGCCATCTC -3’ |
