# Supplementary material for: Prediction of enzymatic pathways by integrative pathway mapping
Source: eLife. 2018 Jan 29;7:e31097. doi: 10.7554/eLife.31097 (PMC5788505; doi:10.7554/eLife.31097)
Supplement: Supplementary file 8. [file elife-31097-supp8.docx]

| **Gene** | **Oligo** | **Sequence (5’-3’)** |
| --- | --- | --- |
| ***Hi*UxuA**  **(Uniprot ID P44488)** | HI0055_qPCR_Fwd | CGTACCTGTTGCCCAAGAAA |
|  | HI0055_qPCR_Rev | CGGAACGTACGCCATAAGAA |
| ***Hi*UxuR**  **(Uniprot ID P44487)** | HI0054_qPCR_Fwd | GCATTGGAGACAAACTCCCTAC |
|  | HI0054_qPCR_Rev | CTACATAAACGCCTGACC |
| ***Hi*GulD**  **(Uniprot ID Q57517)** | HI0053_qPCR_Fwd | CAAGTGGTCGTATGGCTGAA |
|  | HI0053_qPCR_Rev | AGCATATCCAATGCCTCCTC |
| ***Hi*Gul*P***  **(Uniprot ID P71336)** | HI0052_qPCR_Fwd | TCAACTTGGCGGTGAAGA |
|  | HI0052_qPCR_Rev | TAAGGCCATTCCCAAACTCC |
| ***Hi*GulQ**  **(Uniprot ID P44484)** | HI0051_qPCR_Fwd | TATTCCTGCGTTGCCTGA |
|  | HI0051_qPCR_Rev | CCTGTAGCTGGCATTTGTTG |
| ***Hi*GulM**  **(Uniprot ID P44483)** | HI0050_qPCR_Fwd | GCTGGAGCATCCATCACTAA |
|  | HI0050_qPCR_Rev | TCCCTCCTCTTAATCCCACA |
| ***Hi*KdgK**  **(Uniprot ID P44482)** | HI0049_qPCR_Fwd | AGTGCGGTAAGAATGGTGCT |
|  | HI0049_qPCR_Rev | AAAGAATCACCAGCCGAGGT |
| ***Hi*UxuB**  **(Uniprot ID P44481)** | HI0048_qPCR_Fwd | TTCGAGGCTGCAGACAAAGT |
|  | HI0048_qPCR_Rev | GATTATTCCCACCAGCACCA |
| ***Hi*KdgA**  **(Uniprot ID P44480)** | HI0047_qPCR_Fwd | TGCGTGCAAATAGACCTGAC |
|  | HI0047_qPCR_Rev | CCCAGGAGTAACCACAAA |
|  | Hflu_ref_Fwd | TACGGTATTCGTGGGTGGTT |
|  | Hflu_ref_Rev | GTGCAGCTTCACGGTCATC |
