# Supplementary material for: Prediction of enzymatic pathways by integrative pathway mapping
Source: eLife. 2018 Jan 29;7:e31097. doi: 10.7554/eLife.31097 (PMC5788505; doi:10.7554/eLife.31097)
Supplement: Supplementary file 9. [file elife-31097-supp9.docx]

| **Enzyme (Uniprot #)** | **Oligo** | **Sequence (5’-3’)** |
| --- | --- | --- |
| ***Hi*Gul*P***  **(Uniprot ID P71336)** | Del_HI0052_arm1fwd | GTCAGGATCCAAGAAGTGCCTTATCCCGAGAAAAGTG |
|  | Del_HI0052_arm1rev | CCTGCAGGCATGCAAGCTTAACTTTCTCCTTATTGATTATGTTTTTTAGAAAACTGC |
| ***Hi*Gul*P***  **(Uniprot ID P71336)** | Del_HI0052_arm2fwd | GATGCTCGATGAGTTTTTCTAATTAATAAGATCCCCTATGTTCAATTTTATAAATTGC |
|  | Del_HI0052_arm2rev | GTCGAAGCTTGCTTGGAGGAATAACAACTGAAATAATACC |
| ***Hi*Gul*P***  **(Uniprot ID P71336)** | Kan_OL_delHI0052_fwd | GCAGTTTTCTAAAAAACATAATCAATAAGGAGAAAGTTAAGCTTGCATGCCTGCAGG |
|  | Kan_OL_delHI0052_rev | GCAATTTATAAAATTGAACATAGGGGATCTTATTAATTAGAAAAACTCATCGAGCATC |
| ***Hi*GulD**  **(Uniprot ID Q57517)** | Del_HI0053_arm1fwd | CTGAGGATCCTTGACGAATATCAGATAAAG |
|  | Del_HI0053_arm1rev | CCTGCAGGCATGCAAGCTTTTTTAGACTCCTTATATGCCATTGACAGAATAAAATTAC |
| ***Hi*GulD**  **(Uniprot ID Q57517)** | Del_HI0053_arm2fwd | GATGCTCGATGAGTTTTTCTAAAAAACATAATCAATAAGGAGAAAGTTATGAAATC |
|  | Del_HI0053_arm2rev | CTTACTGCAGTTAATTTTTTGGACTACATCC |
| ***Hi*GulD**  **(Uniprot ID Q57517)** | Kan_OL_delHI0053_fwd | GTAATTTTATTCTGTCAATGGCATATAAGGAGTCTAAAAAAGCTTGCATGCCTGCAGG |
|  | Kan_OL_delHI0053_rev | GATTTCATAACTTTCTCCTTATTGATTATGTTTTTTAGAAAAACTCATCGAGCATC |
